# Supplementary material for: Proteome biology of primary colorectal carcinoma and corresponding liver metastases
Source: Neoplasia. 2021 Nov 9;23(12):1240–51. doi: 10.1016/j.neo.2021.10.005 (PMC8591399; doi:10.1016/j.neo.2021.10.005)

# Additional Files

**Supplementary Figure 1. Correlation analysis of technical replicates of primary colorectal carcinoma and liver metastasis samples from seven patients.**
Correlation analysis of the measured intensities of primary colorectal carcinoma (A) and liver metastasis (B) tissue. Pearson correlation coefficients are shown in the bottom left corner and illustrated as ellipses in the upper right corner. Correlation for the two technical replicates of the same patient sample is highlighted in green.


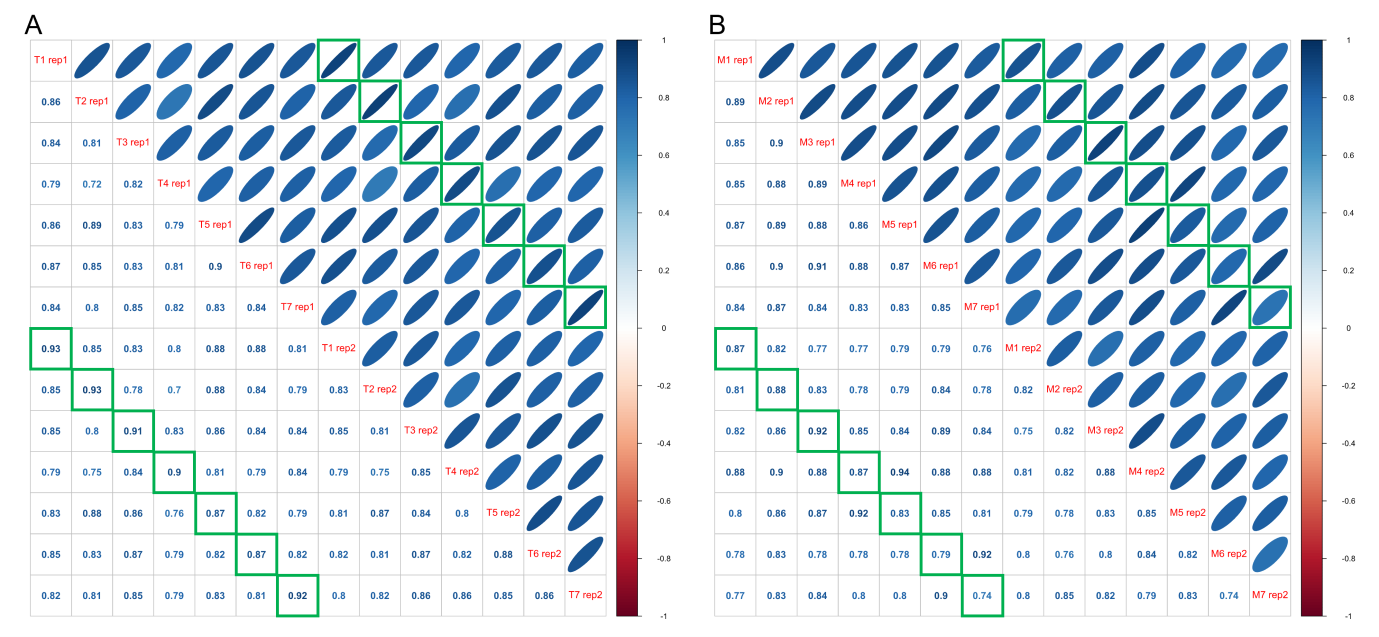


**Supplementary Figure 2. Overview of identified and quantified proteins in non-dissected primary CRC and liver metastases.**
The bar chart shows the number of identified and quantified proteins in primary colorectal cancer (blue) and liver metastases (yellow) samples from n = 7 patients.


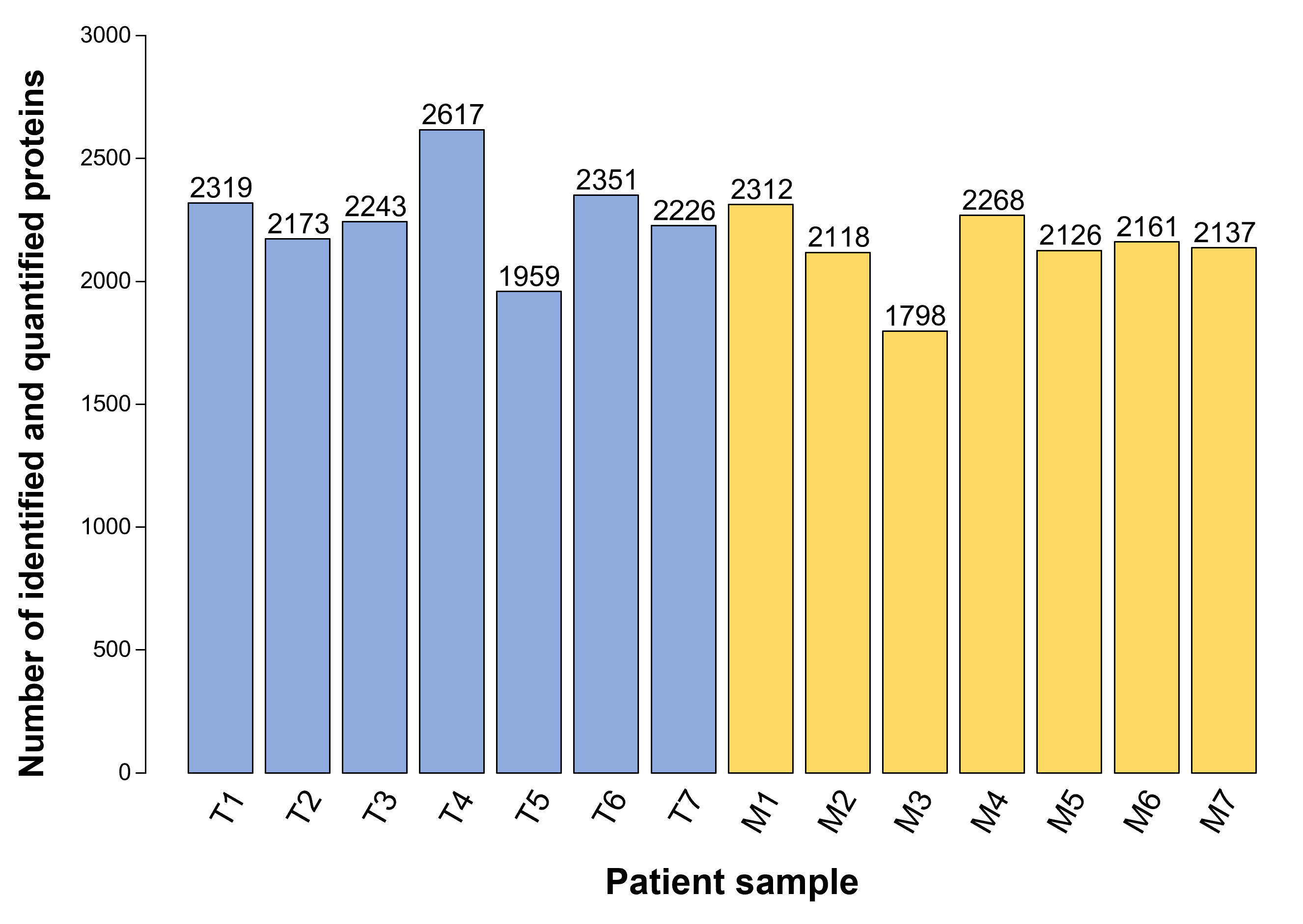


**Supplementary Figure 3. Unsupervised analysis of non-dissected primary CRC and liver metastases.**
Proteins that were at least qualified in 4 out of the 14 samples were used for unsupervised principal component analysis (A) and hierarchical clustering (B).


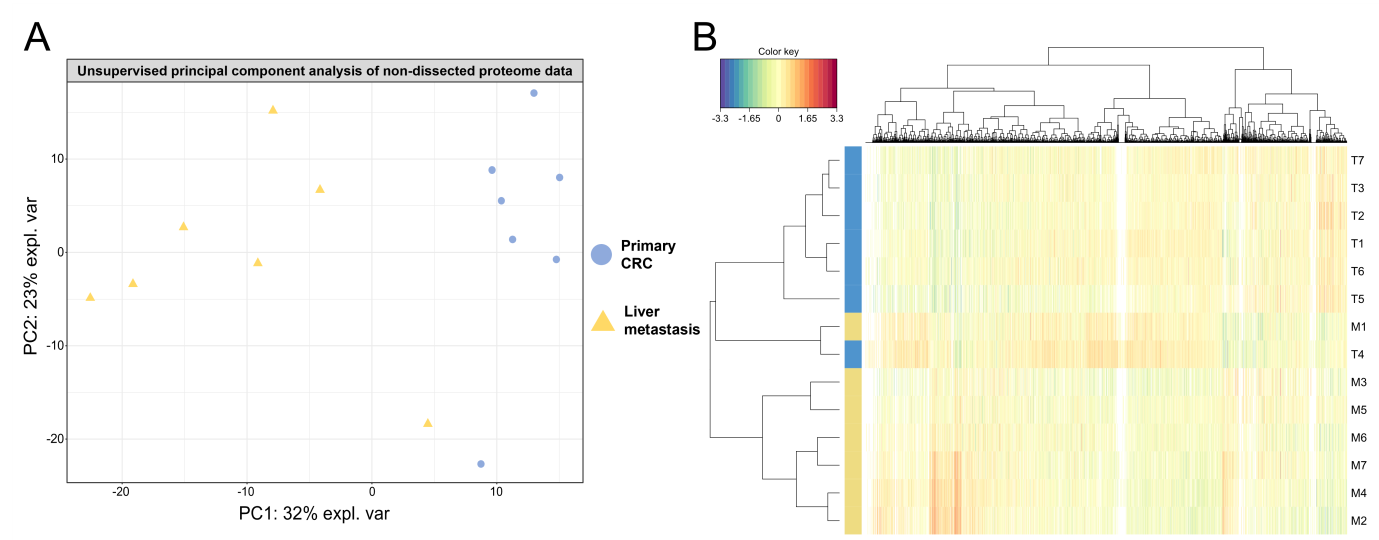


**Supplementary Figure 4. Statistical analysis of non-dissected primary CRC and liver metastases of n = 7 patients.**
A) Volcano plot showing proteins with their respective -log10 adjusted p-value and the log2 fold change for the comparison of liver metastases against primary CRC tissue from seven patients. Of the 2461 proteins, 105 were significantly more abundant in liver metastases, whereas 100 proteins were significantly more abundant in primary CRC (adjusted p-value < 0.05). B) Gene ontology (GO) analysis of the significantly dysregulated proteins shows upregulated biological processes for each tumor tissue.


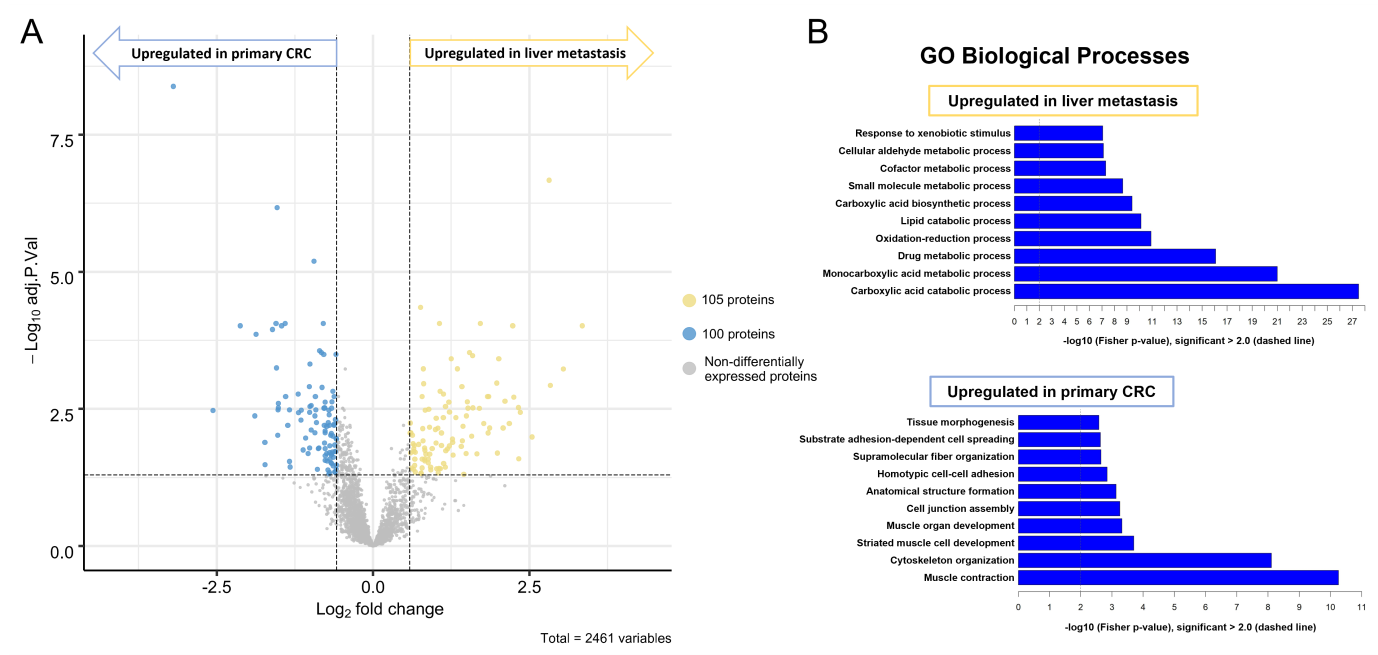


**Supplementary Figure 5. REACTOME analysis of primary CRC and liver metastases of n = 7 patients.**
REACTOME analysis of the significantly dysregulated proteins in A) the dissected and B) the non-dissected proteome data. The dot plot shows the affected pathways and the number of dysregulated genes as well as the adjusted p-value.


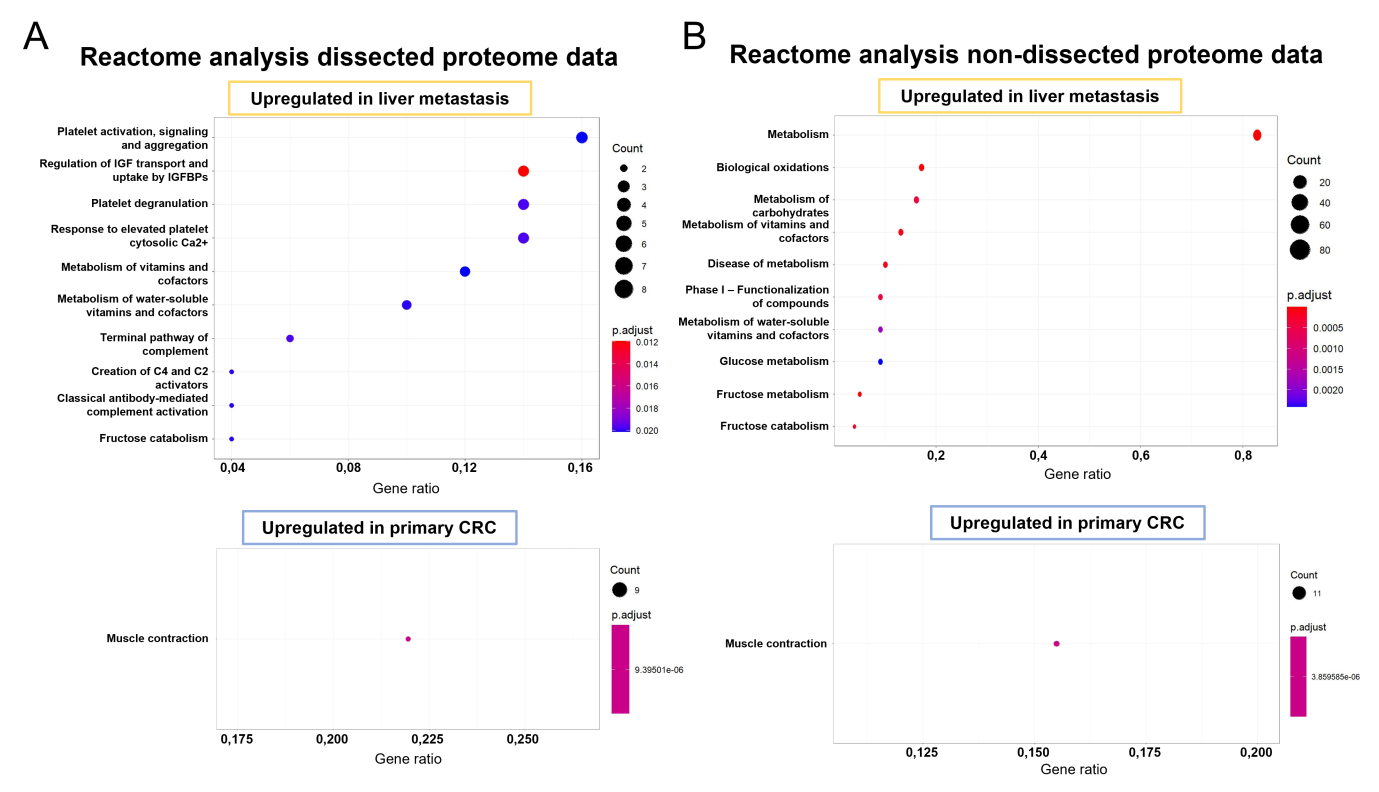


**Supplementary Figure 6. Statistical analysis highlighting proteins of interest in the comparison of primary CRC and their corresponding liver metastases.**
Volcano plot showing proteins with their respective -log10 adjusted p-value and the log2 fold change for the comparison of liver metastases against primary CRC tissue from seven patients. Of the 2697 proteins, 57 were significantly more abundant in liver metastasis, whereas 51 proteins were significantly more abundant in primary colorectal tumors (adjusted p-value < 0.05). Proteins of interest (POI) that were used for follow-up investigation using either Immunohistochemistry (IHC) or proliferation assays are highlighted in red.


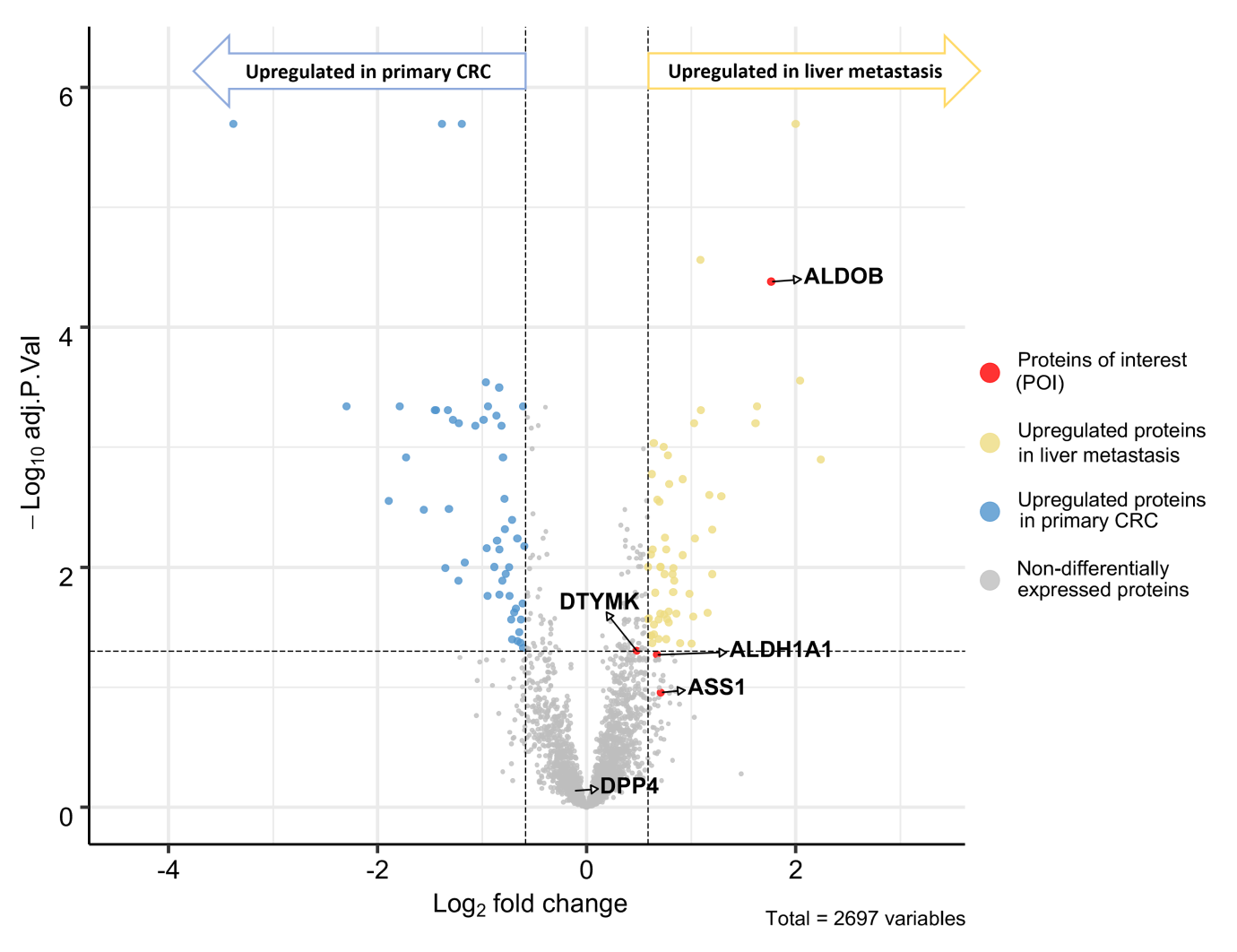

Supplement: Supplementary file 1 [file mmc1.docx]
